# Editorial: Nanomaterials for targeted delivery of therapeutic and imaging agents

**DOI:** 10.3389/fcell.2022.978690

**Published:** 2022-08-05

**Authors:** S. Chockalingam, Gopinath Packirisamy, Ramasamy Paulmurugan

**Affiliations:** ^1^ Department of Biotechnology, National Institute of Technology Warangal, Warangal, Telangana, India; ^2^ Centre for Nanotechnology, Indian Institute of Technology, Roorkee, Uttarakhand, India; ^3^ Stanford University School of Medicine, Palo Alto, CA, United States

**Keywords:** nanomaterials, theranostics, imaging, targeted delivery, Polymer nanoparticles

Nanomedicine has been a growing field over the last couple of decades, and the development of new platforms for improving disease diagnosis, therapies, and drug formulations has been constantly evolving. The rapid clinical translation of the current Covid-19 mRNA vaccines is also in part due to the application of nanotechnology mediated gene delivery. Nanomaterials, while helping to develop targeted delivery of therapeutics, also serve as imaging agents/contrasts for monitoring the disease status *in vivo* using various imaging modalities. Nano-agents like gold nanoparticles, nano-diamonds, and carbon nanotubes work as excellent drug carriers while helping in intracellular tracking of delivered agents. To highlight the application of nanomaterials in therapy and diagnostic imaging (theragnostic), we invited articles related to this topic from established investigators. We received a total of seven manuscripts, including four original research manuscripts and three review articles, among of which six of them that were related to nanomaterials in theranostic applications were accepted and assembled as a special issue.

**FIGURE 1:**
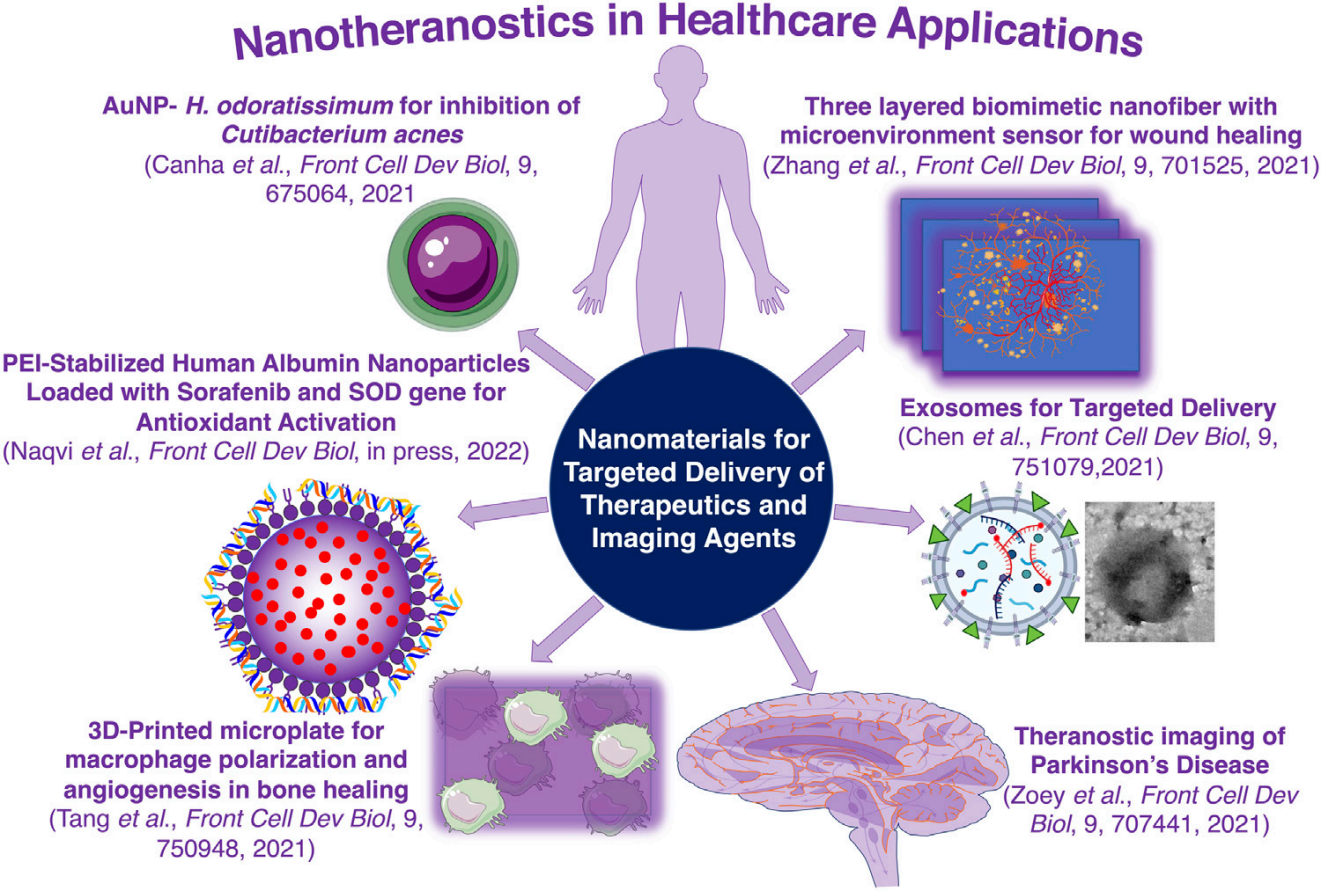
Schematic illustration of theranostic nanoparticles of various types in healthcare applications covered in this special issue.

As shown in the figure (Figure 1), the original research manuscript by De Canha et al. highlighted the preparation of gold nanoparticles by incorporating extract of *Helichrysum odoratissimum* as a potential nanoformulation for effective inhibition of *Cutibacterium acnes,* a commensal that causes acne in human skin, adhesion without showing any antibacterial activity (De Canha et al.). This serves as a useful formulation for preventing bacterial disease, and at the same time overcomes the possibility of developing a drug resistant bacterium, a common problem associated with the use of antibiotics for treating bacterial diseases. Similarly, Naqvi et al., has developed a polyethyleneimine (PEI) stabilized human serum albumin (HSA) nanoparticles for simultaneous delivery of a small molecule antioxidant sulforaphane (SF) and an antioxidant responsive SOD1 gene for improving the antioxidant property of cells (Naqvi et al.). This type of delivery application will have a significant impact on the viability of cells in cell transplantation therapy, especially stem cell transplantation in cardiac therapy and β-cell transplantation in diabetes, by improving the survival of cells by enhancing the intracellular antioxidants level. In the same line of health care associated nanomaterial applications, the research manuscript by Zhang et al., has shown the preparation of a three-layered smart dressing biomimetic nanofiber membrane integrated with a microenvironment sensor (Zhang et al.). This novel dressing material for wound healing applications was prepared by cross-linking β-cyclodextrin-containing gelatin methacryloyl (GelMA + β-cd) within hydrogel. The integrated hydrogel in this biomimetic nanofiber helped wound healing by increasing the expression of vascular endothelial growth factor (VEGF) through hypoxia-inducible factor-1α (HIF-1α) to promote neovascularization.

In general, nanomaterials synthesized at different conditions can result in particles with different shapes and sizes that are responsible for their distinct physical and chemical properties, which prove beneficial in a wide variety of applications (Dreaden et al., 2012). Multifunctional nanosystems exhibiting the dual properties of drug delivery to targeted areas while simultaneously helping in imaging are called theranostic nanoparticles. Theranostic nanoparticles with their intrinsic magnetic and optical properties or labelled using radionuclides can enhance the imaging efficiency of techniques like magnetic resonance imaging (MRI), positron emission tomography (PET), and optical imaging. For instance, gold nanoparticles functionalized with glucosamine are used as contrast agents in computed tomography (CT) imaging (Silvestri et al., 2016). Theranostic nanoparticles are also shown to be effective in the treatment of Covid-19, cardiovascular diseases, and cancer.

Three-dimensional (3D) printing is currently an emerging technology for making nanomaterial-based health care devices. Recently, 3D-printed microneedles were used for intradermal delivery of antiviral vaccines to achieve strong immune responses and for insulin delivery to achieve long-term functional effect. In this line, the manuscript by Tang et al., adapted a 3D printing using Ti2448 alloys for printing low stiffness plates for bone healing applications (Tang et al.). This system enhanced angiogenesis and osteogenesis by regulating macrophage recruitment and its polarization *via* Piezo1/YAP signaling axis. The use of this material in an orthopedic surgery resulted in recruiting M2 macrophages to the implant site and promoted angiogenesis. In addition, it also promoted osteogenic differentiation through increasing PDGF-BB and BMP-2 secretions and modulating Piezo1/YAP signaling *via* macrophage polarization and related cytokines levels. While the above four research manuscripts highlighted the importance of nanomaterials in healthcare related applications by research results, two other manuscripts extensively reviewed the nanotechnology in theranostic applications. One manuscript extensively discussed the treatment and imaging of Parkinson’s disease. The other manuscript discussed recent advances in exosomes; small nanosized vesicles secreted by the cells as their natural communication system, for drug and small RNA delivery applications in different diseases, including cancer.

Parkinson’s disease (PD) is a common age-related neurodegenerative disorder. A progressive growth of patients with PD is increasing in several developed countries, leading to a tremendous healthcare burden. The aggregation of α-synuclein in the brain is the main cause for the neuronal dysfunction in this disease. There are no successful therapies available, and there are delivery- and toxicity-associated problems that limit the curative application of existing drugs. The blood brain barrier also postulates another major challenge in delivering effective doses of drugs for treating this disease. Nanotechnology, overcoming many of these barriers, has been explored in many drug delivery applications. Recently, several theranostic agents have been developed for treating the disease while monitoring their delivery by imaging. In this regard, a review manuscript by Zoey et al., has provided information on the use of various nanomaterials for theranostic applications in early diagnosis and targeting α-synuclein for improving cognitive functions in patients with PD (Zoey et al.). This manuscript addressed the mechanism of disease development, and the list of diagnostics, therapeutics, and imaging strategies available for targeting PD using both preclinical and clinical imaging modalities. This review also briefly discussed the BBB related issues in therapeutic deliveries, and how antibody conjugated nanoparticles (SPION) can improve delivery across BBB.

Finally, a review manuscript by Chen et al., extensively discussed the story around exosomes, a small membrane vesicle secreted by cells. This review mainly discusses about the biogenesis, biological functions, and loading therapeutic cargos and their applications in various diseases using exosomes (Chen et al.). Exosomes are nanovesicles (generally with a size of 30–150 nm) secreted by cells loaded with cellular cargos as a natural communication system between cells. Based on the source cells, the loaded cellular cargos can be highly variable, which determine the biological properties of the exosomes. Recently, exosomes from various cells have been extensively evaluated for their biological functions in various diseases and applications in drug deliveries. Overall, this special issue provides a consolidated knowledge on the theranostic value of various nanomaterials and the associated technologies in healthcare applications. Although, nanotechnology has been extensively studied, the limited success of applications in the clinic is mainly due to the immune system and the associated adverse effect. Hence, recently a large amount of focus has been diverted towards soft nanomaterials (polymer nanoparticles and cell membrane vesicles), which possess flexibility to engineer to overcome immune system by immune evasion, and the exosomes with their inherent biomimetic effect. The development of biomimetic theranostic nanoparticles with selective immunomodulatory property will have tremendous role in various healthcare applications in the future.
